# Supplementary material for: The Evolution and Characterization of the RNA Interference Pathways in Lophotrochozoa
Source: Genome Biol Evol. 2024 May 7;16(5):evae098. doi: 10.1093/gbe/evae098 (PMC11114477; doi:10.1093/gbe/evae098)
Supplement: evae098_Supplementary_Data [file evae098_supplementary_data.zip › Supplementary_materials.zip!/selected_miRNAs/NoGe_selected_miRNAs/novel_miRNA_families_pdf_files/NMRB01000267.1_Notospermus_geniculatus_strain_Ushimado_scaffold267,_whole_genome_shotgun_sequence_8670.pdf]

| | | |
|---|---|---|
| Provisional ID | : | NMRB01000267.1_Notospermus_geniculatus_strain_Ushimado_scaffold267,_whole_genome_shotgun_sequence_8670 |
| Score total | : | 46 |
| Score for star read(s) | : | 3.9 |
| Score for read counts | : | 41.4 |
| Score for mfe | : | 1.2 |
| Score for randfold | : | 0 |
| Score for cons. seed | : | -0.6 |
| Total read count | : | 93 |
| Mature read count | : | 50 |
| Loop read count | : | 0 |
| Star read count | : | 43 |

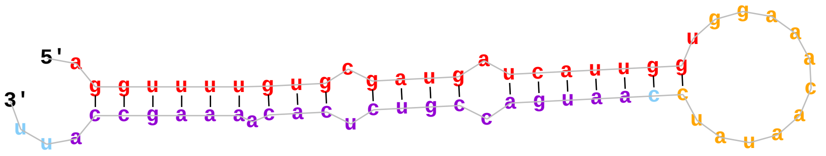


Genome coordinates: NMRB01000267.1 312048-312107

pre-miRNA sequence:
AGGTTTTGTGCGATGATCATTGGTAGGTTTTGTGCGATGATCATTGGTGGAAACAATATCCAATGACCGTCTCACAAAAGCCA

Mature sequence: AGGTTTTGTGCGATGATCATTGGT

BLAST results for the pre-miRNA sequence against the *Lineus longissimus* genome assembly:

| Scaffold | Start | Stop | Identity | e-value |
|---|---|---|---|---|
| OU343000.1 1 | 6146103 | 6146045 | 93.220 | 4.70e-17 |

```
Nge_novel_miRNA                    AGGTTTTGTGCGATGATCATTGGTGGAAACAATATCCAATGACCGTCTCACAAAAGCCA
Llo_genome                         AGGTTTTGTGCGATGATCATTGGTGGTGACAATATTCAATGACCGTCTTACAAAAGCCA
```
